# Healthcare workers’ perceptions about the use of mobile health technologies in public health facilities in Lagos, Nigeria

**DOI:** 10.1177/20503121231224568

**Published:** 2024-02-12

**Authors:** Oluwatobi Shekoni, Synne Iversen, Gabriela J Diaz, Anders Aune, Peter Odion Ubuane, Zainab Imam, Beate André

**Affiliations:** 1 Department of Public Health, Norwegian University of Science and Technology (NTNU), Trondheim, Norway; 2 Picterus AS, Trondheim, Norway; 3 Department of Pediatrics, Lagos State University Teaching Hospital (LASUTH), Ikeja Lagos, Nigeria

**Keywords:** mHealth, neonatal jaundice, *Picterus*, healthcare workers, neonatal health, Nigeria, sub-Saharan Africa

## Abstract

**Background:**

Mobile health has enormous potential in healthcare due to the increasing use of mobile phones in low- and middle-income countries; its effective deployment, uptake, and utilization may result in improved health outcomes, including a reduction in neonatal deaths. However, there is a suboptimal uptake of mobile health technologies among healthcare workers in low-resource settings like Nigeria, which are often context-specific.

**Objective:**

To investigate healthcare workers’ perceptions of mobile health technologies in public health facilities in Lagos, Nigeria.

**Method:**

A qualitative study was conducted, and data were collected through six focus group discussions with 26 healthcare workers (doctors, nurses, and community health extension workers) from three public health facilities in Lagos, Nigeria. The collected data were analyzed using a thematic approach, where themes and subthemes were created.

**Results:**

Although the participants acknowledged that mobile health enhances patient–provider communication and saves time, they identified altering of healthcare workers’ routine practices, information overload, power and network failure, skepticism, lack of trust, and concerns over diagnostic accuracy as potential barriers to its uptake.

**Conclusion:**

Addressing healthcare workers’ perceptions of mobile health technologies may enhance the deployment and uptake of such solutions in Nigeria and similar low-resource settings. Developers and implementers of such can use them to create new or enhance existing mobile health solutions to better meet the needs and requirements of healthcare workers in low- to middle-income health settings, such as Lagos, Nigeria.

## Introduction

Mobile health (mHealth) is defined by the World Health Organization as “medical and public health practice supported by mobile devices, such as mobile phones, patient monitoring devices, personal digital assistants, and other wireless devices.”^1^ mHealth is crucial in healthcare delivery, particularly in low- and middle-income countries (LMICs),^2,3^ and is an essential component in the promotion of Universal Health Coverage.^4^ mHealth technologies have great potential to make healthcare more effective, accessible, and affordable.^5^ For example, short-text message (SMS) appointment reminders are widely used in LMICs^6,7^ to encourage patients to attend their appointments.^8^ Previous research discovered that SMS reminders effectively increased clinic attendance for urban pediatric residents with high no-show rates.^8^ With SMS, people living in remote areas can now receive health-related information.^6^ Similarly, a program implemented in 2010 by the Clinton Health Access Initiative in Nigeria used SMS printers to speed up the return of HIV/AIDS test results for infants; this enabled healthcare facilities to receive and print test results without the use of computers or the Internet and resulted in a significant reduction in turnaround time and loss-to-follow-up.^9^ A study conducted by Benski et al. in rural Madagascar implemented the mHealth system called Pregnancy and Newborn Diagnostic Assessment (PANDA) to improve antenatal care for pregnant women. With the deployment of PANDA, they discovered that women began to arrive earlier for their first antenatal care (ANC) visit.^10^

Mobile phone availability and use have grown particularly rapidly in LMICs, where having a phone is frequently more common than having access to clean water and electricity.^11^ In 2021, nearly 15 billion mobile devices were used worldwide, which is expected to rise to 18 billion by 2025.^12^ The modern smartphone is thus an excellent technology for the widespread delivery of healthcare,^3^ especially sub-Saharan Africa, where it is expected to account for around two-thirds of total Sub-Saharan African connections by 2025.^13^

The development of phone-based software applications (apps) is one of the areas of mHealth that is rapidly expanding due to the increasing use of mobile devices such as smartphones.^14,15^ Numerous health screening apps are now available to assist healthcare workers (HCWs) in screening, diagnosing, and treating patients.^14,16^ An example is *Picterus*^®^ Picterus AS, Trondheim, Norway, a smartphone app that uses digital images and a calibration card to estimate the serum bilirubin levels of babies with jaundice.^17^ Thus, this app has the potential to assist HCWs, and even parents, in detecting this potentially catastrophic disease^18^ early so that appropriate remedial care can be given or sought before major brain injury occurs.^17,19,20^ Such an app is most needed in sub-Saharan Africa, with the world’s highest number of deaths from neonatal jaundice. Nigeria is responsible for many preventable neonatal mortalities,^21^ and pediatricians in Nigeria have highlighted neonatal jaundice as a priority illness requiring global health action.^22^ Reducing neonatal deaths continues to be a global priority, expressed as SDG target three.^23^ According to the WHO, many neonatal deaths in LMICs could be prevented with cost-effective solutions like mHealth technologies.^24,25^

Despite the introduction of mHealth technologies and their benefits,^5^ surprisingly few initiatives have successfully scaled up or incorporated mHealth into health programs in developing countries beyond the pilot stage.^26–28^ Integrating new mHealth technologies is a complex process^29^; its uptake can be limited by several factors,^5^ resulting in low utilization in LMICs.^29^ Sundin et al. identified such factors to include challenges with cost, technology, management, and technology users.^5,29^ Furthermore, user acceptability is regarded as essential for implementing new mHealth technologies.^30^ Paduano et al. sought to assess HCW acceptance of a mHealth system utilized during ANC visits in Tanzania. The results showed that the system was widely accepted by the majority of HCWs due to its ease of use.^31^ Exploring user acceptability is thus crucial in implementing innovative mHealth technologies since it can give potential implementers valuable insight into factors influencing users’ motivation to utilize the technology.^30^ According to Nilsen et al., one of the major challenges to the deployment and uptake of mHealth technology is user resistance, which can be related to both the technology and resistance to change.^32^ Several factors have been associated with resistance to change, including negative attitudes^33^ and a lack of knowledge, beliefs, trust, and acceptance.^34^

HCWs play a crucial role in adopting and sustaining mHealth.^35^ Their perceptions can disclose important information about their acceptability and motivation to use various technologies in practice.^36,37^ The current study thus aimed to explore, qualitatively via focus group discussions (FGDs), the perception of HCWs—doctors, nurses, and community health extension workers—in public healthcare facilities in Lagos State, Nigeria, regarding mHealth technologies, using *Picterus*^®^ as a demonstrated model.

## Methods

### Study design

We employed a qualitative research approach using FGDs.

### Study population and setting

We invited HCWs, namely doctors, nurses, and community health extension workers (CHEWs) from public primary, secondary, and tertiary health facilities across Lagos State—the most populous city in Nigeria and one of the most populous megacities in the world.

CHEWs are healthcare providers trained to provide basic promotive, preventive, and selected curative health services at primary healthcare centers (PHCs) in an accessible and equitable manner to all sections of the population, including to mothers and children at the local community level, especially in rural areas lacking more skilled health workers like doctors and nurses. Nigeria’s three-tier healthcare system comprises primary, secondary, and tertiary facilities. PHCs are patients’ primary contact points, operating at the community level and providing basic preventive, promotive, curative, and rehabilitative services.^38^ Secondary health facilities are hospitals, mainly general hospitals with outpatient specialist clinics, to which patients are referred from PHCs to receive more specialized care. Tertiary health facilities provide treatment for specific specialties, rare diseases, or situations in which it is challenging to find diagnostic or therapeutic facilities or require scarce combinations of resources.^38^

Nigeria is among the countries in Africa with the most extensive stock of human resources for health.^39^ Compared to the sub-Saharan African average where the doctor-population ratio is 15 per 100,000 population, the Nigerian doctor-population ratio is 38.9 per 100,000 population. Furthermore, the nurse/midwife population ratio of the country is 148 per 100,000 population. Despite these numbers, Nigeria still has a severe and ongoing lack of HCWs to meet the demands of its population, particularly in some parts of the country.^39^

All HCWs invited to our study offered care to children in their hospital, some exclusively. HCWs working in private healthcare facilities were excluded.

### Sample size and sampling technique

Inclusion criteria include the following:

1. HCWs (doctors, nurses, and CHEWs) working at a public hospital in Lagos State. The Nigerian population primarily uses public health facilities and the use of mHealth technology is low in this setting.
2. HCWs working with pediatric patients. This is because during the FDGs a demonstration of a mHealth app used for pediatric patients was done.

Exclusion criteria include the following:

1. HCWs working in private healthcare facilities were excluded from this study.

Based on the size of the local government area’s population, the facility for recruitment was selected. We sampled the facilities in the most populous local government areas. A total of 26 HCWs were purposively selected from three public health facilities in Lagos, Nigeria, and were grouped according to their profession, namely doctors, nurses, and CHEWs, to reflect varied perceptions across HCWs.

### Ethical approvals

Ethical approval was obtained from the Health Research and Ethics Committee of the Lagos State University Teaching Hospital (LREC) in Nigeria on October 18, 2021 (Ref. No.: LREC/06/10/1688). The Lagos State Primary Healthcare Board, the Lagos State Health Service Commission, and the heads of the respective facilities and departments also gave written permission to conduct the study. All participating HCWs also gave both written and verbal informed consent to the audio-recorded FGDs.

### Recruitment process and data collection

**Figure 1.**
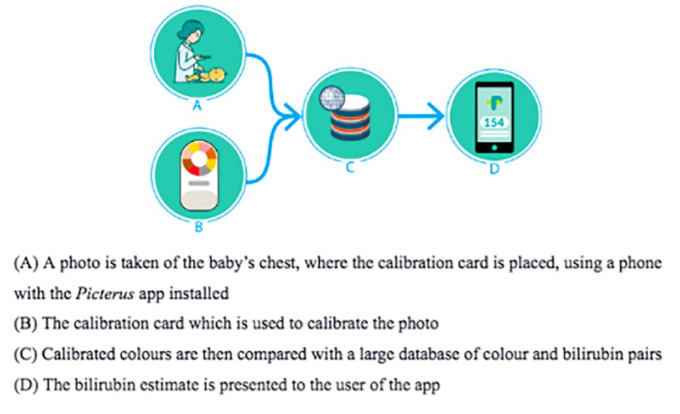
Demonstration of the *Picterus* app. Source: Aune et al., 2020.

Before the data collection, we designed a semi-structured interview guide consisting of open-ended questions, which were pilot-tested with seven participants. HCWs were approached and informed of the study at each health facility through their unit heads, and a suitable date and venue were fixed. The FGD occurred from facility to facility between January and February 2022. The lead authors and one of the supervisors conducted the FGDs using the interview guide (available on Mendeley Data: doi:10.17632/tzvmbxzwxk.1). All sessions were audio-recorded using a SONY IC™ Voice Recorder, Konan Minato-ku, Tokyo, Japan, and field notes were made during each FGD. Also, during the FGDs, the mHealth app *Picterus*^®^ was used as an example of a clinically applicable mHealth solution by demonstrating its use on a mannequin (Figure 1).

Each FGD lasted between 43 and 59 min; each group consisted of three to six HCWs. All FGDs were held in person in a room at each health facility. The conversations were conducted in English.

### Data analysis

The audio recordings were transcribed verbatim and uploaded to the software program NVivo for the coding procedure and the remainder of the data analysis.^40^ FGDs were analyzed using Braun and Clarke’s six-step thematic analysis (TA) method.^41^ The coding and the rest of the analysis were done by two of the researchers. A complete coding strategy was employed, which included coding all information pertinent to the research question.^42^ To identify themes within the data, an inductive approach was utilized. Thematic saturation was achieved in this study when participants’ responses were repetitive and no new themes appeared.^42,43^ The data collection was then stopped.

## Results

### Participant demographics

A total of 26 HCWs from three public health facilities participated in the FGDs: 10 doctors, 13 nurses, and 3 CHEWs. Most of them were female, and their years of experience varied (Table 1).

**Table 1.** Demographics of the participants.

Profession	Gender	Age range	Years of experience
Doctor	2 males 8 females	25–50 years	3 months–16 years
Nurse	13 females	25–60 years	6 months–34 years
Community health extension workers (CHEWs)	1 male 2 females	31–38 years	2–3 years

### Themes

During TA of the data, two main overarching themes emerged: (1) perceived benefits that promote the use of mHealth and (2) perceived barriers that limit the use of mHealth. Each aspect and its accompanying subthemes are detailed in Table 2. Selected transcribed statements are also shown below as texts.

**Table 2.** Schematic overview of the themes identified through thematic analysis.

Main overarching themes	Subthemes
Perceived benefits that promote the use of mHealth	Facilitating access to health-related information Enhances patient–provider communication - Online consultations - Reaching more people with mHealth Saving time
Perceived barriers that limit the use of mHealth	Altering healthcare workers’ routine practices Skepticism, lack of trust, and concerns over diagnostic accuracy Information overload Power and network failure

#### Perceived benefits that promote the use of mHealth

Three subthemes linked to the first overarching themes were revealed: facilitating access to health-related information, enhancing patient–provider communication, and saving time.

##### Facilitating access to health-related information

Most participants highlighted how mHealth technologies facilitate access to health-related information. They pointed out how access to information could positively impact the health of the whole population:

*You know. . . Even people that don’t work in the health setting can search about diseases using a mobile phone*. (FGD 2, CHEW 2)

*. . .With the increased information, people are more informed which has helped reduce mortality. [Information] would help [patients] to make early health-related decisions and has also helped people to have a good health-seeking behavior. . .* (FGD 6, Doctor 2)

#### Enhancing patient–provider communication

##### Online consultations

Furthermore, participants opined that mHealth enables online consultations:

*. . .I don’t need to see a doctor physically. . . There are some things that I can do with these digital skills online without seeing my doctor. . . mHealth can be easily accessible anywhere I am. I don’t need to drag myself to the hospital*. (FGD 3, Nurse 2)

##### Reaching more people with mHealth

Several participants underlined how access to mobile phones could help them reach more people who struggle to attend healthcare facilities due to factors such as distance, transportation, and inadequate infrastructure:

*More people can be reached really. . . Because where some people live, it’s actually really difficult to come out to town. . . If they have a mobile phone, they can call and chat with the healthcare workers. . .* (FGD 6, Doctor 1)

#### Saving time

Most participants discussed the benefits of using mHealth technologies with respect to time-saving. As the FGDs included a demonstration of the mHealth app *Picterus*, several of them used the app as an example to convey their message:

. . .*Picterus will reduce the time for withdrawing the blood when taking a regular blood test. . . It will reduce that time to screening, to carry out the procedure. . . With what you did within a second [when demonstrating the Picterus app]. . . The lab can spend more than one hour. . . With Picterus, it can go faster and reduce time consumption*. (FGD 2, CHEW 1)

Furthermore, the participants believed that mHealth technologies would facilitate communication and collaboration between healthcare facilities in several ways, as exemplified by the following statement:

. . .*We have to liaise from both public and private hospitals and laboratories to get blood. . . With mHealth, you can quickly call any lab and ask, ‘Do you have this blood type?’ Instead of moving around [looking for it]. I remember there was a time when we were using bikes. . . Motorcycles and scooters to find blood. . .* (FGD 6, Doctor 6)

#### Perceived barriers that limit the use of mHealth

Four subthemes linked to the second overarching theme were revealed: altering healthcare routine practices; skepticism and lack of trust; information overload; and power and network failure.

##### Altering HCWs’ routine practices

Participants voiced concern that mHealth technology would displace them and make their jobs less intellectually challenging:

*[mHealth] could be a disadvantage to the medical personnel by making us lazy. . .*

*We would no longer want to seek in-depth information. . . It just makes you do things monotonously. The medical personnel are no longer challenged to think, unlike when these [mHealth technologies] were not there. It will no longer make us use the higher center of the brain*. (FGD 4, Doctor 2)

Moreover, several participants raised concerns about the necessity of learning to type on a mobile device after being used to writing by hand:

*It’s not easy to implement something new. . . A place where a lot of people have been used to writing. . . I sometimes hear [health workers] complain about having to type. . . People are not used to it*. (FGD 6, Doctor 4)

##### Skepticism, lack of trust, and concerns over diagnostic accuracy

Most of the participants emphasized the importance of trust in deploying various mHealth technologies, particularly those designed to facilitate screening or diagnosis:

*. . .If the healthcare worker does not believe that the mHealth technology is accurate, it will not be easily adopted*. (FGD 4, Doctor 3)

Some participants reported hearing or reading about inaccurate mHealth applications to test and diagnose. After hearing or reading about these experiences, several participants developed distrust toward such technologies:

*For instance, recording patients’ data and using mHealth for accessing information is okay but using mHealth for tests is not okay*. (FGD 2, CHEW 3)

Furthermore, some worried that the accuracy of mHealth technologies, such as the *Picterus*^®^ app, could be influenced by external factors peculiar to their practice settings:

. . . *The places where these technologies are produced may not put some things into consideration. . . Like if you keep these devices in a certain temperature, I can bet you that it will malfunction because some of the devices have sensors. . . It is physics. . . Whoever brings these technologies will have to look at how these aspects can be addressed*. (FGD 4, Doctor 5)

##### Information overload

Several participants expressed concerns about the negative effects associated with patients having access to a lot of information with new mHealth technologies:

*I remember one particular patient that went home and started searching online. Then he was seeing all the wrong information. . . The Internet page just gave all the information, so the patient became anxious and ended up in the emergency room for psychiatric evaluation. Too much information can lead to anxiety and other emotional trouble*. (FGD 6, Doctor 5)

##### Power and network failure

The majority of the participants acknowledged that prevalent power and network failure or connectivity issues would pose difficulty in implementing mHealth in Nigerian public healthcare institutions:

*There are challenges with the power supply. . . Technology thrives on the constant adequate power supply*. (FGD 6, Doctor 5)

*Sometimes if there’s no network connectivity. If I need to access mHealth, what do I do? If there is nothing I can do, I’ll be paralyzed for that moment when there is no network. . . So, if there is a technical issue, fault, or anything, I won’t be able to access mHealth. . .* (FGD 4, Doctor 2)

## Discussion

In this qualitative study of the perception of HCWs working and offering childcare services in three public healthcare facilities across Lagos State, Nigeria, on the use of mHealth in healthcare delivery, we identified potential benefits and barriers to its uptake and utilization. To enhance the discussion, we illustrated the use of mHealth with *Picterus*^®^, a novel smartphone app that estimates bilirubin levels. The participating HCWs identified the benefits of mHealth utilization to include enhancing access to health-related information, facilitating patient–provider communication, and saving time while noting the challenges to include information overload, power and network failure, skepticism and lack of trust, and altering healthcare workers’ routine practices.

### The balance of information

The HCWs in our study viewed mHealth as beneficial because it improved access to health-related information. This increases patient involvement in their health and encourages better healthcare-seeking behaviors. Improved health-seeking performance may translate to improved preventive measures and earlier detection and treatment of illnesses. Consequently, health outcomes, including morbidity and mortality, may improve. According to Vo et al., patients valued educational applications that provided them with health knowledge, making them more aware and giving them more control over their health situation, thereby empowering them.^44^ Similarly, Paduano et al. found that using mHealth technology served as an effective tool in delivering health education to pregnant women during ANC visits.^31^

However, our participants noted that having a wealth of information available is not always advantageous. They were concerned that information overload could cause anxiety, sometimes with negative consequences. According to Bawden and Robinson, information anxiety may be caused by information overload.^45^ Therefore, it is crucial that HCWs engage their clients with the information obtained online and guide them regarding what materials to consume.

### The balance of physical and online consultations

Participants believed online consultations would reduce their workload and save patients time. HCWs believed that by utilizing mHealth technologies, specifically via mobile phones, more people, including those living in remote areas, would have increased access to healthcare services. Similarly, Hampshire et al. emphasized the importance of mHealth in remote areas, particularly during emergencies so that people can obtain health assistance quickly.^46^ In addition, HCWs believed online consultations could limit the face-to-face connection between patients and HCWs. This was especially helpful during the height of the COVID-19 pandemic.

### Saving time

mHealth was perceived as a time-saving solution for HCWs and patients. Time-consuming and laborious procedures and duties may be completed more quickly and efficiently with mHealth technologies. mHealth apps like *Picterus*^®^ can save time and improve patient outcomes. In Nigeria, a laboratory-based biochemical assay of serum bilirubin is the mainstay diagnostic modality for neonatal jaundice, often beset by long turnaround time resulting in delayed treatment and consequences like death, bilirubin encephalopathy, and cerebral palsy. On the contrary, the use of *Picterus*^®^ can serve as a screening tool for estimating bilirubin levels within 2 min without the need for invasive and painful sampling; this, in turn, may translate to earlier treatment and better outcomes.^17^ Also, medical experts can be contacted via mobile devices (mobile consultation), allowing quicker and easier decision-making.

Although mHealth technologies have the potential to save time, Paduano et al. found that the ANC visits lasted longer when mHealth technology was used. Despite this, HCWs perceived the mHealth system as an improvement in the quality of service rather than an additional workload.^31^

### Accepting changes in routine processes and practices

Several HCWs feared losing their clinical skills to mHealth. Concerns were expressed about the use of mHealth technologies may make the HCWs “lazy” or make their work monotonous and less interesting. In other words, using mHealth could be seen as a threat to their current practices.^47^ Similarly, a previous study discovered that nurses feared losing skills because of technological advancements.^48^ On the other hand, Fleming emphasizes the connection between fear and resistance to change, which can stem from a fear of losing control.^49^ It is acknowledged that deploying new technologies modifies current health practices and routines,^50^ a significant obstacle to successfully implementing mHealth technologies.^51^ As highlighted in the study by Walter and Lopez,^52^ if the user of a product perceives that the intention is to restrict autonomy, the user may not want to utilize the product. Acceptance is necessary for scaling and maintaining mobile technologies in healthcare.^52–54^ People are often very comfortable with current practices and old behaviors, according to Haslam and Pennington.^55^ Although these old behaviors may not be productive, many people resist changing because changing could make them uncomfortable and threatened.^49,55^

### Trust and acceptance

HCWs expressed concerns about the accuracy and reliability of using mHealth devices for screening and diagnosis; a valid concern to avoid errors of misdiagnosis with potential harm to patients. Thus, mHealth devices need to be validated and calibrated in settings outside of where they were developed.^56^ Sadly, research has revealed the existence of mHealth applications that endanger patient safety and may harm users.^56^ HCWs with prior knowledge or experience with inaccurate mHealth devices were less likely to use technological tools in healthcare. As a result, some HCWs would prefer to continue to use a time-consuming, inconvenient traditional method over quicker and simpler ones. Although skepticism is seen as a barrier to using mHealth technology, it has the potential to benefit society. There may be advantages to being skeptical of new technologies, as this skepticism stems from concerns about the technology’s accuracy and adaptability to a new environment. In our study, HCWs were concerned about the adaptability of mHealth technologies to their environment. As an illustration, they were skeptical about adopting *Picterus*^®^, an app developed initially using the Norwegian population, for use among the Nigerian population. Hence, mHealth developers should partner with local researchers in high-burden settings to conduct context-specific validation and calibration studies before possible market deployment.

### Infrastructural and technological limitations

Nigeria still faces enormous electricity shortages and outages, as well as reasonably frequent Internet connectivity issues. Our participants thus rightly emphasized that the effective utilization of many modern mHealth technologies, such as the Picterus app, may be limited by unstable Internet infrastructures. One of the top three barriers to implementing mHealth technologies in healthcare is inadequate infrastructure components such as power supply and Internet connectivity.^7^ According to a WHO report, poor infrastructure is also a major barrier to the widespread adoption of mHealth technology.^1^ Without good Internet connectivity, the use of mHealth can be frustrating and time-consuming, and HCWs may be forced to abandon the application in favor of conventional methods.

### Limitations

We acknowledge that our study may not represent the varied opinions, perceptions, and contexts across healthcare settings in Lagos and Nigeria. However, we achieved thematic saturation after no additional emerging themes were found. Although we did not include other categories of HCWs such as pharmacists and laboratory scientists whose services could also be deployed with mHealth, we involved frontline healthcare providers across the primary, secondary, and tertiary levels of care, thus providing a reasonable level of assessment. Non-inclusion of HCWs in the private sector limits generalization beyond public healthcare in Lagos. Furthermore, although the interview guide was pilot-tested, it was not validated, which is another limitation of this study. Further studies are thus needed to explore the perceptions of a broader range of HCWs, including those in private settings and outside Lagos.

## Conclusion

The current study provided insight into the benefits that may promote the use of mHealth and the barriers that could limit its usage from the perspectives of public HCWs in primary, secondary, and tertiary health facilities in Lagos. The perceived benefits that promote the use of mHealth technology include facilitating access to health-related information, enhancing patient-provider communication, and time savings. By contrast, perceived barriers that may limit its use include altering healthcare routine practices, skepticism and lack of trust, information overload, and power and network failure. The findings of this study can be used to address potential barriers to the deployment of new mHealth technologies or improve the acceptability of existing ones, which could translate to improved access to healthcare and health outcomes.

## Supplemental Material

sj-docx-1-smo-10.1177_20503121231224568 – Supplemental material for Healthcare workers’ perceptions about the use of mobile health technologies in public health facilities in Lagos, Nigeria Click here for additional data file. Supplemental material, sj-docx-1-smo-10.1177_20503121231224568 for Healthcare workers’ perceptions about the use of mobile health technologies in public health facilities in Lagos, Nigeria by Oluwatobi Shekoni, Synne Iversen, Gabriela J Diaz, Anders Aune, Peter Odion Ubuane, Zainab Imam and Beate André in SAGE Open Medicine

sj-docx-2-smo-10.1177_20503121231224568 – Supplemental material for Healthcare workers’ perceptions about the use of mobile health technologies in public health facilities in Lagos, Nigeria Click here for additional data file. Supplemental material, sj-docx-2-smo-10.1177_20503121231224568 for Healthcare workers’ perceptions about the use of mobile health technologies in public health facilities in Lagos, Nigeria by Oluwatobi Shekoni, Synne Iversen, Gabriela J Diaz, Anders Aune, Peter Odion Ubuane, Zainab Imam and Beate André in SAGE Open Medicine
