# Supplementary material for: Healthcare workers’ perceptions about the use of mobile health technologies in public health facilities in Lagos, Nigeria
Source: SAGE Open Med. 2024 Feb 12;12:20503121231224568. doi: 10.1177/20503121231224568 (PMC10860469; doi:10.1177/20503121231224568)
Supplement: sj-docx-2-smo-10.1177_20503121231224568 – Supplemental material for Healthcare workers’ perceptions about the use of mobile health technologies in public health facilities in Lagos, Nigeria [file sj-docx-2-smo-10.1177_20503121231224568.docx]

**mHealth – Focus Group Guide**

Start by welcoming everyone and thank you for participating. Start by introducing yourself (moderator).

*Tell them that you have now started the recording.*

Some short information and ground rules during the FG: (tell the participants)

● The discussion will take approximately 45 mins to 1 hour

● Try to talk to each other, rather than (just) answering the moderator ● We are seeking your thoughts and opinions, meaning there are no right and wrong answers

● One person speaking at a time: Please try to not talk over the top of each other, this will make the transcription part difficult - and we do not want to miss any of your comments

● We will only use first names in this discussion, and we will not use any of your names in the report

Let us have a round of introduction: first name(?), age, position/work.

**Starting question**

● Today's topic is Mobile Health. What are your thoughts about it?

● What is the first thing that comes to mind when I say the word “mHealth/mobile health?”

**Mobile Health**

● What do you know about mobile health?

● Have you ever used any form of mobile health before? If so, please tell me about your experiences.

*Probes: Positive/Negative? Did it work out? Why/Why not? Issues/Concerns?* ● In what ways are you aware of mobile health being used in your hospital setting?

● What factors do you think must be in place for acceptance of mobile health? *Probes:*

*- More knowledge about it?*

*- Evidence/research?*

*- Support from stakeholders?*

*- Cost Effective solutions?*

● Some mobile health interventions work out, while others do not - why do you think that is?

*Probes:*

*- Money? Too expensive?*

*- Internet connection?*

*- Too advanced?*

*- Skepticism?*

● In what way do you think mobile health could support the current health systems?

**Picterus Jaundice App**

● What are your thoughts about this idea/app demonstration?

● How are you currently assessing neonatal jaundice?

*Probes: Does it work out? Why? Why not?*

● How would you use it in your setting?

● How would your patient/patient guardian respond to the use of this app? ● Who is responsible for the adoption of this app?

● What do you think are the pros and cons of this product/app?

● In what other area can this mHealth tool be used in your health facility? *Probes: Would you give an example?*

● In what way do you think this app could affect your practice, either negatively or positively?

*Probes: Could you please elaborate?*

**Closing**

● Is there anything other than the already discussed questions that you would like to share or talk more about?

Thank you for participating in this discussion. *Stop the recording*
